# Physiological Responses in Sons of Alcoholics

**Published:** 1997

**Authors:** Peter R. Finn, Alicia Justus

**Affiliations:** Peter R. Finn, Ph.D., is an associate professor of psychology and director of the Clinical Science Training Program and Alicia Justus is director of the Biobehavioral Alcohol Research Laboratory in the Department of Psychology at Indiana University, Bloomington, Indiana

**Keywords:** children of alcoholics, son, physiological stress, psychological stress, AOD use susceptibility, risk factors, hereditary factors, electroencephalography, autonomic nervous system, behavioral and mental disorder, skin, AODE (alcohol and other drug effects), cortisol, adrenocorticotropic hormone, heart function, AOD intoxication, AOD tolerance, AOD impairment, reinforcement, endorphin, amounts of AOD use, AOD sensitivity, comparative study, literature review

## Abstract

Researchers have differentiated sons of alcoholics (SOA’s) from sons of nonalcoholics (non-SOA’s) on various measures of physiological activity that appear to be related to the SOA’s increased vulnerability to developing alcohol problems. This article summarizes major findings in the literature and discusses the implications of risk-related physiological characteristics for the future development of alcohol problems. SOA’s tend to show signs of physiological activity associated with anxiety states, such as increased heart rate in response to stressful stimuli. Studies also demonstrate that SOA’s differ greatly from non-SOA’s in their response to alcohol. Drinking alcohol dramatically reduces SOAs’ reactivity to both stressful and nonstressful stimuli. Additionally, SOA’s appear to be less sensitive to alcohol’s intoxicating and impairing effects. However, studies also suggest that some SOA’s may experience more of alcohol’s rewarding effects for a brief period after drinking. Increased stress-dampening and reduced responsiveness to alcohol’s negative effects also appear to predict the development of future alcohol problems and may reflect important vulnerabilities in SOA’s.

Studies indicate that sons of alcoholics (SOA’s) differ from sons of nonalcoholics (non-SOA’s) on a range of physiological activity measures, and the differences appear to be related to the SOA’s increased vulnerability to developing alcohol problems. Researchers hope that investigating such differences will offer insights into the factors that may be responsible for SOA’s higher risk for alcohol abuse.

This article summarizes the major findings on differential physiological responses from studies conducted to date. Unfortunately, most of these studies examine physiological activity only in SOA’s and exclude daughters of alcoholics. The reason typically given for this exclusion is that some data suggest that sons, but not daughters, of alcoholics are at elevated risk for alcoholism.^1^ Nonetheless, it is encouraging that the National Institute on Alcohol Abuse and Alcoholism (NIAAA) now funds studies on children of alcoholics only if the research design includes both sons and daughters.

Studies of SOA’s usually assess physiological responses, such as brain wave (electroencephalographic [EEG]) activity,^2^ autonomic nervous system^3^ responses (e.g., heart rate or perspiration), body sway,^4^ and hormone levels, in reaction to three general scenarios: (1) nonstressful, resting conditions; (2) stressful conditions; and (3) after drinking alcohol. Although SOA’s are not a homogeneous population, certain physiological responses often characterize them. This article reviews research findings for each of these three distinct scenarios.

## Physiological Activity of SOA’s in Nonstressful, Resting Conditions

The overall picture emerging from studies of physiological responses under nonstressful, resting conditions indicates that compared with non-SOA’s, SOA’s experience a greater preponderance of high-frequency EEG activity and increased autonomic reactivity in response to nonstressful stimuli, such as hearing a simple tone over headphones. Both of these general results suggest a higher level of anxiety or tension in SOA’s compared with non-SOA’s. Because studies in this area are somewhat inconsistent, however, findings should be interpreted cautiously (Finn in press).

Several studies report that SOA’s exhibit higher levels of high-frequency EEG activity than do non-SOA’s (for a review, see Finn in press). For example, Gabrielli and colleagues (1982) observed that preadolescent sons of alcoholic fathers experienced a greater percentage of high-frequency EEG activity compared with non-SOA’s. Similar patterns of increased high-frequency EEG activity have been reported in adult SOA’s (Ehlers and Schuckit 1991), alcoholics (Gabrielli et al. 1982), and their relatives (Pollock et al. 1995). Although the precise meaning of these results is unclear, higher levels of high-frequency EEG activity have been associated with elevated levels of anxiety (Gabrielli et al. 1982).

The majority of studies report no differences between SOA’s and non-SOA’s when assessing autonomic activity at baseline (i.e., before presentation of a stimulus). However, studies examining autonomic responses to nonstressful stimuli suggest that at least some SOA’s are more reactive to such stimuli (Finn et al. 1990; cf. Finn in press). SOA’s have been found to perspire more (i.e., have greater skin conductance) than non-SOA’s in response to nonstressful tones presented over headphones at a loudness level similar to that of a passing car (Finn et al. 1990). In addition, SOA’s often continue to respond to these types of tones longer than do non-SOA’s (i.e., SOA’s are slower to habituate). Similar patterns of increased reactivity and longer habituation to nonstressful stimuli have been observed in people with anxiety disorders, suggesting that this pattern of physiological response in SOA’s may indicate a propensity toward anxiety-related problems.

## Physiological Activity of SOA’s in Stressful Conditions

**Figure 1:**
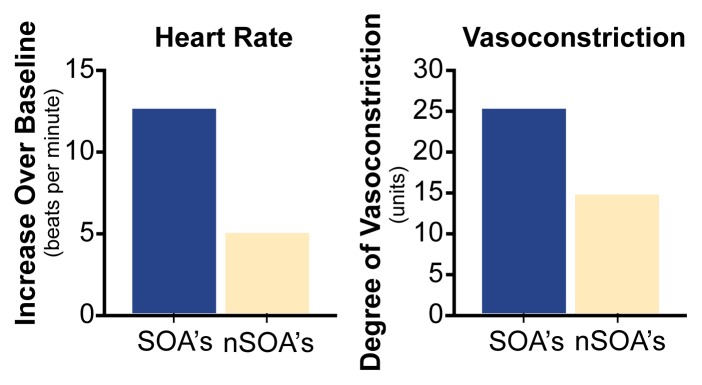
Stress reactivity when sober. Sons of alcoholics (SOA’s) and sons of nonalcoholics (nSOA’s) differ in their response to electric shock in measures of heart rate (left) and vasoconstriction in the hand (right). SOA’s experienced both a higher increase in heart rate and a greater degree of vasoconstriction compared with nSOA’s, indicating a higher stress level. NOTE: Blood vessel constriction (i.e., vasoconstriction) is measured in arbitrary analog digital computer measurement units. SOURCE: Finn et al. 1990.

Several studies measuring autonomic nervous system activity indicate that SOA’s with anxious tendencies react strongly (i.e., they are hyperreactive) to stressful stimuli, such as receiving a mildly painful electric shock (for a review, see Finn in press). For example, Finn and colleagues (1990) reported that compared with non-SOA’s, young adult SOA’s ages 18 to 25 show higher heart rates and greater vein constriction (i.e., vasoconstriction) in the hand while awaiting an electric shock, as illustrated in figure 1. These patterns of increased heart rate and vasoconstriction are classic manifestations of a typical cardiovascular response to stress, suggesting that the SOA’s in this study were more stressed by the prospect of receiving an electric shock than were the non-SOA’s. Increased heart rate reactivity to stress also has been reported in adolescent SOA’s during performance of a mental arithmetic task (Harden and Pihl 1995). Anxious traits (e.g., nervousness and excessive worrying) and a family history of anxiety disorders appear to be related to increased stress-induced cardiovascular reactivity in SOA’s (Finn in press; Harden and Pihl 1995). In theory, this pattern of hyperreactivity in SOA’s may make them more vulnerable to using alcohol excessively to self-medicate in an effort to reduce their high levels of stress reactivity (Finn et al. 1990). This theory is especially appealing given the fact that SOA’s appear to show increased sensitivity to alcohol’s stress-reducing effects, as discussed in the next section.

Some SOA’s exhibit high levels of antisocial traits and generally disruptive behavior (Finn et al. 1997). Research suggests that these SOA’s may actually be less responsive than non-SOA’s to the threat of negative events such as a painful electric shock. Finn and colleagues (1994) found that compared with non-SOA’s, some SOA’s show weaker skin conductance responses to tones that are followed by electric shocks. This pattern of low reactivity to the threat of pain also has been found in psychopathic (i.e., antisocial) individuals (cf. Finn in press). Theoretically, people who do not become upset (i.e., are not reactive) about impending negative events are less likely to inhibit behaviors that often lead to negative consequences. Finn (in press) speculates that SOA’s who are underreactive to the threat of punishment may not learn from their mistakes as readily as do non-SOA’s and thus seem more likely to experience alcohol-related problems, such as missing work, failing an exam, or being arrested for driving while intoxicated (Finn et al. 1997). Before firm conclusions can be made about the association between antisocial behavior and physiological activity in SOA’s, however, more research is required to replicate and extend the findings of Finn and colleagues (1994).

## Physiological Activity of SOA’s After Drinking Alcohol

By far, the majority of studies examining physiological activity in SOA’s examine the effects of alcohol on various measures of physiological functioning. These studies reveal three major results:

- SOA’s show smaller cardiovascular reactions to stress than do non-SOA’s after consuming moderate to high doses of alcohol (i.e., three to four drinks);
- Compared with non-SOA’s, SOA’s experience less body sway and weaker hormone responses to alcohol when assessed during resting conditions; and
- After drinking alcohol, SOA’s also experience more of the physiological changes associated with pleasurable effects compared with non-SOA’s, although only immediately after drinking.

Researchers disagree on the interpretation of some of these results (e.g., SOAs’ reduced cardiovascular response to stress after drinking alcohol). Current theories suggest that SOA’s may demonstrate enhanced responsiveness to alcohol as their blood alcohol levels rise just after drinking but reduced responsiveness after blood alcohol levels begin to decline as they “sober up.”

### Alcohol’s Effect on Cardiac Reactivity

**Figure 2:**
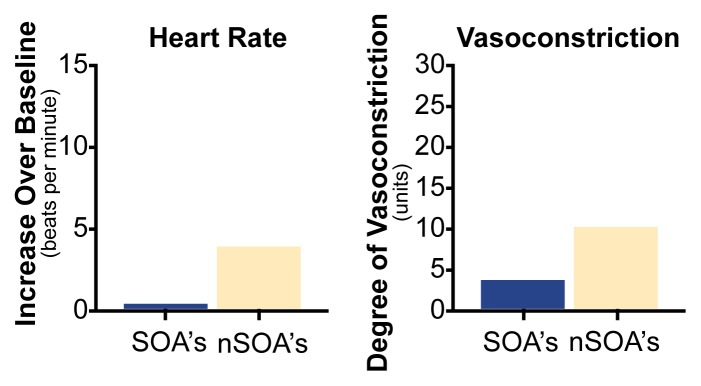
Stress reactivity after drinking. Increases in heart rate (left) and vasoconstriction (right) in response to electric shock were measured in sons of alcoholics (SOA’s) and sons of non-alcoholics (nSOA’s) after they consumed two to three alcoholic beverages. Compared with nSOA’s, SOA’s experienced a smaller increase in both measures, indicating a lower degree of stress reactivity. A comparison of these results with those shown in figure 1 reveals a dramatic drop in stress reactivity in SOA’s after drinking. NOTE: Blood vessel constriction (i.e., vasoconstriction) is measured in arbitrary analog digital computer measurement units. SOURCE: Finn et al. 1990.

Many studies have reported that SOA’s show a substantial decrease in stress reactions on measures of both cardiac activity (e.g., Finn et al. 1990; Conrod et al. 1995) and muscle tension (Conrod et al. 1995; Finn et al. 1990) after drinking alcohol. Following the consumption of three to four drinks of alcohol, which results in blood alcohol levels between 0.075 and 0.13 percent, SOA’s seem to be calm and undisturbed when faced with significant stressors, such as electric shock. In contrast, non-SOA’s demonstrate only modest decreases in stress reactions after drinking. Figure 2 displays a typical pattern of cardiac reactivity to stress in SOA’s and non-SOA’s after they drink alcoholic beverages. Comparison of figures 1 and 2 illustrates the dramatic reduction in reactivity in SOA’s before and after drinking in contrast with the relatively minor reduction in non-SOA’s.

Finn and colleagues (1990) also found that SOA’s were significantly less reactive to nonstressful stimuli after drinking alcohol. Thus, the general pattern of reduced reactivity after drinking may predispose SOA’s to consume alcohol excessively to help them cope, especially in stressful situations (Finn in press). In fact, recent data suggest that increased sensitivity to the stress-reducing effects of alcohol can predict higher levels of alcohol consumption (Finn in press).

### Alcohol’s Effect on Body Sway and Hormone Responses

In addition to studies suggesting that SOA’s are more sensitive to alcohol’s stress-reducing effects, another body of literature indicates that SOA’s may be more tolerant than non-SOA’s to alcohol’s intoxicating effects. SOA’s, when matched with non-SOA’s exhibiting the same drinking patterns, consistently report being less intoxicated than non-SOA’s after consuming the same amount of alcohol (see, for example, O’Malley and Maisto 1985; Schuckit et al. 1996). SOA’s also appear to be significantly less impaired on measures of body sway than non-SOA’s after drinking two to three alcoholic beverages (see, for example, Schuckit et al. 1996).

**Figure 3:**
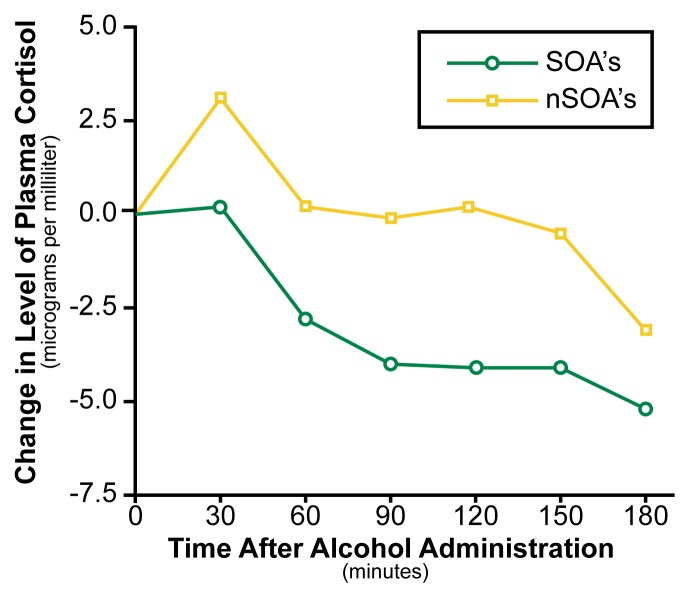
Plasma cortisol levels of sons of alcoholics (SOA’s) and sons of nonalcoholics (nSOA’s) at 30-minute intervals after consuming 2.5 drinks of alcohol. Although alcohol consumption typically increases cortisol production, SOA’s produce less cortisol after drinking compared with nSOA’s, providing further evidence of SOA’s greater tolerance to some alcohol effects. SOURCE: Schuckit et al. 1996.

Schuckit and colleagues (1996) also found that SOA’s produce less cortisol than non-SOA’s do after drinking two to three drinks (figure 3), as well as less adrenocorticotropic hormone (ACTH). The typical increases in cortisol and ACTH that occur after drinking reflect alcohol’s effect on specific brain areas controlling the release of these hormones and perhaps also a mild stress response to the presence of alcohol as a toxin in the system. The results of Schuckit and colleagues suggest that SOA’s simply have reduced central nervous system responses to alcohol. These results replicate findings from an earlier study (see Schuckit 1995) using a different sample of SOA’s and non-SOA’s matched for drinking habits. Both studies imply that SOA’s drinking the same amount over a similar period of time as non-SOA’s appear either to be more innately tolerant to certain alcohol effects or to develop tolerance more rapidly.

Furthermore, research indicates that the increased tolerance of SOA’s represents a significant risk factor for developing alcohol problems. Schuckit (1995) notes that a weaker response to alcohol at age 20 (i.e., a need to drink more alcohol to experience an effect) is a strong predictor of alcohol problems at age 28. Similar results have been reported by Volavka and colleagues (1996), who showed that smaller changes in EEG activity (i.e., increased tolerance) after youth in late adolescence consumed two to three drinks was associated with more alcohol problems and consumption 10 years later.

Why should increased innate tolerance lead to alcohol problems? The basic theory is that people learn to regulate their drinking by monitoring alcohol’s effects on their level of functioning, then adjust their intake based on the effects they experience. Typically, a person stops or at least slows down the rate of drinking when he or she begins to feel impaired. The experience of being impaired or intoxicated thus serves as a feedback mechanism that contributes to the regulation of alcohol intake. Failure to experience the normal impairing effects of alcohol, however, may lead to a breakdown in this regulatory system, which may be reflected in the increased tolerance exhibited by SOA’s. It is possible that SOA’s drink more alcohol partly because they do not experience the usual negative feedback of intoxicated feelings or psychomotor impairment (i.e., increased body sway). Subsequently, they are at greater risk for developing alcohol-related health, occupational, or legal problems or other complications attributable to their high alcohol intake.

### Alcohol’s Rewarding Effects

A handful of studies suggest that some SOA’s show enhanced responses to alcohol on measures of hormonal (Gianoulakis et al. 1996), EEG (Cohen et al. 1993), and cardiac activity (for a review, see Finn in press) that are theoretically linked to pharmacological reward mechanisms (i.e., positive reinforcement). For example, Gianoulakis and colleagues (1996) found that SOA’s experience greater increases in blood levels of a naturally occurring opiatelike chemical (i.e., beta-endorphin) than non-SOA’s do after consuming moderate doses of alcohol (defined in this study as approximately three drinks), but not low doses (i.e., one drink). Although beta-endorphin activity in the brain has been associated with the basic reward mechanisms of a variety of drugs (Finn in press; Gianoulakis et al. 1996), it is unclear whether blood levels of beta-endorphin levels are associated with brain reward mechanisms.

Research also suggests that some SOA’s exhibit greater increases in heart rate over baseline after consuming alcohol when compared with non-SOA’s (Finn in press; Finn et al. 1990). Alcohol-induced increases in heart rate are thought to reflect the initial stimulatory effect associated with rising blood levels of alcohol (Finn in press) and have been associated with increases in plasma beta-endorphin levels (cf. Finn in press).

Similarly, Cohen and colleagues (1993) reported that after two to three drinks, SOA’s show greater enhancements of low-frequency EEG activity compared with non-SOA’s. This effect occurs only immediately after consuming an alcoholic beverage, however, while blood alcohol levels rise. Furthermore, Cohen and colleagues (1993) reported that the enhancement of low-frequency EEG activity disappears relatively quickly in SOA’s compared with non-SOA’s, providing further evidence of SOAs’ greater tolerance to alcohol. Previous studies have associated drug-induced increases in low-frequency EEG activity with euphoria (cf. Finn in press). The 1993 study by Cohen and colleagues indicates that this heightened effect is short-lived, persisting only as the SOAs’ blood alcohol levels rise, and then is replaced by significantly lower levels of low-frequency EEG activity as blood alcohol levels decline, which might produce unpleasant feelings (i.e., dysphoria). These results suggest that SOA’s who experience this effect would be motivated to continue drinking in order to maintain the euphoric experience of enhanced low-frequency EEG activity and avoid the dysphoric experience of a major reduction in low-frequency EEG activity while “sobering up.”

Taken together, studies of alcohol’s effects on SOA’s suggest that SOA’s may be more sensitive to alcohol’s “rewarding” effects when their blood alcohol levels are rising, but less sensitive to impairment and intoxication at peak and descending blood alcohol levels. The relationship among euphoria, plasma beta-endorphin, and low-frequency EEG activity is purely speculative, however. Alcohol-induced increases in plasma beta-endorphin, low-frequency EEG activity, or heart rate responses have not been associated with increased alcohol-induced euphoria in SOA’s.

## Summary and Conclusions

The general pattern emerging from the studies cited in this article implies that SOA’s tend to exhibit levels of physiological activity that indicate increased tension and stress reactivity. In addition, the studies clearly demonstrate that SOA’s respond differently to alcohol than do non-SOA’s on several dimensions. For example, drinking alcohol reduces stress reactivity in SOA’s to a much greater extent than in non-SOA’s. SOA’s also seem less sensitive to alcohol’s intoxicating effects, as evidenced by reduced body sway, cortisol secretion, and subjective reports of intoxication. Such reduced impairment after drinking has been found to predict future alcohol problems and appears to reflect a significant vulnerability to alcoholism. Some studies also suggest that SOA’s may experience more of alcohol’s rewarding effects for a brief period after drinking compared with non-SOA’s. In addition, research suggests that some SOA’s may exhibit enhanced beta-endorphin and EEG responses to alcohol that are hypothetically related to positive reinforcement mechanisms.

Finally, it is emphasized that SOA’s are not all alike and, in fact, represent a rather heterogeneous group. Finn and colleagues (1997) found three distinct patterns, or subtypes, of vulnerability to alcoholism in the families of SOA’s: (1) low levels of psychopathology and moderate levels of alcoholism; (2) high levels of antisocial personality, violence, and alcoholism; and (3) high levels of depression, mania, anxiety disorders, and alcoholism. Finn and colleagues (1997) also report that SOA’s from each family type vary in terms of personality and adjustment characteristics. It is possible that different aspects of physiological activity and sensitivity to alcohol may be associated with distinct vulnerability patterns transmitted within the various types of alcoholic families. Future research is needed to further understand the associations among measures of physiological activity that distinguish SOA’s from non-SOA’s, specific types of vulnerabilities transmitted in alcoholic families, and the development of alcohol problems.

## Footnotes

1 The term “alcoholism” as used in this article refers to the specific criteria for a psychiatric diagnosis of alcohol dependence or abuse given in the American Psychiatric Association’s *Diagnostic and Statistical Manual of Mental Disorders*, *Fourth Edition*. The term “alcoholic” refers to a person who meets these criteria.

2 EEG activity consists of brain waves that typically vary in frequency from 0.5 to 30 cycles per second (hertz [Hz]). Lower frequencies generally are thought to indicate lower arousal or a lower level of activation in the cerebral cortex, whereas higher frequencies are thought to reflect increased activation. High-frequency EEG activity, which is often referred to as “beta activity,” includes relatively small (i.e., low-amplitude) brain waves varying from 12 to 30 Hz in frequency. (Some researchers also include frequencies between 10 and 12 Hz in this category.) Low-frequency EEG activity, also referred to as “slow alpha activity,” includes brain waves varying from 8 to 10 Hz. Low-frequency alpha waves also have a much larger amplitude than high-frequency beta waves. Relaxation and meditation have been found to increase the amount of slow alpha activity. Studies also suggest that reductions or deficits in slow alpha activity are associated with greater brain activation and anxiety states (cf. Finn in press).

3 The autonomic branch of the nervous system helps regulate basic body functions, including heart activity, digestion, sweating, and respiration. In threatening, emergency, or stressful circumstances, the autonomic nervous system increases some of the physiological processes it controls (e.g., heart rate, sweating, and constriction of the veins in the extremities) to prepare the body for “fight or flight” coping responses.

4 Body sway reflects the extent to which a person’s body moves back and forth or side to side when he or she is asked to stand perfectly straight on a level platform. Body sway is measured in terms of the degree the platform tilts if a person shifts his or her weight slightly off center. In most people, alcohol seriously impairs the ability to maintain balance (i.e., psychomotor impairment), as evidenced by increased body sway.
